# Assessment of Wall Elasticity Variations on Intraluminal Haemodynamics in Descending Aortic Dissections Using a Lumped-Parameter Model

**DOI:** 10.1371/journal.pone.0124011

**Published:** 2015-04-16

**Authors:** Paula A. Rudenick, Bart H. Bijnens, Patrick Segers, David García-Dorado, Arturo Evangelista

**Affiliations:** 1 University Hospital and Research Institute Vall d’Hebron, Universitat Autònoma de Barcelona, Barcelona, Spain; 2 Physense, DTIC, Universitat Pompeu Fabra, Barcelona, Spain; 3 ICREA, Barcelona, Spain; 4 Biofluid, Tissue and Solid Mechanics for Medical Applications, Institute Biomedical Technology, Ghent University, Ghent, Belgium; Tianjin University, CHINA

## Abstract

Descending aortic dissection (DAD) is associated with high morbidity and mortality rates. Aortic wall stiffness is a variable often altered in DAD patients and potentially involved in long-term outcome. However, its relevance is still mostly unknown. To gain more detailed knowledge of how wall elasticity (compliance) might influence intraluminal haemodynamics in DAD, a lumped-parameter model was developed based on experimental data from a pulsatile hydraulic circuit and validated for 8 clinical scenarios. Next, the variations of intraluminal pressures and flows were assessed as a function of wall elasticity. In comparison with the most rigid-wall case, an increase in elasticity to physiological values was associated with a decrease in systolic and increase in diastolic pressures of up to 33% and 63% respectively, with a subsequent decrease in the pressure wave amplitude of up to 86%. Moreover, it was related to an increase in multidirectional intraluminal flows and transition of behaviour as 2 parallel vessels towards a vessel with a side-chamber. The model supports the extremely important role of wall elasticity as determinant of intraluminal pressures and flow patterns for DAD, and thus, the relevance of considering it during clinical assessment and computational modelling of the disease.

## Introduction

**Fig 1:**
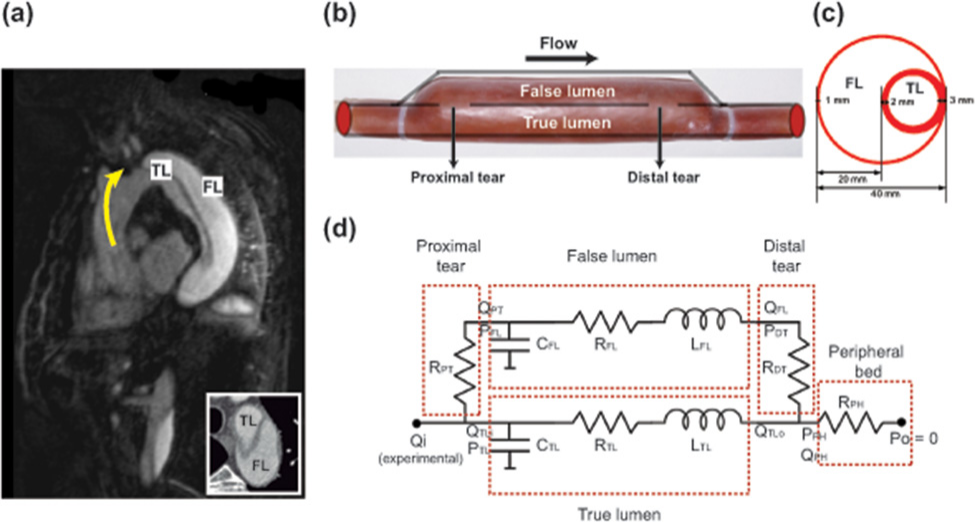
Proposed experimental representation of a clinical aortic dissection and its equivalent lumped-parameter model. (a) Clinical appearance of a descending aortic dissection in the longitudinal plane. Transversal plane showing the distinction between TL and FL (Bottom right) (b-c) Proposed anatomic representation of a descending aortic dissection. Longitudinal diagram of the experimental model (b) and cross-sectional plane of the dissected segment (c). (d) Schema of the lumped-parameter model. The dissected region was modelled as two parallel compartments communicated by resistances (rigid tears). Dashed lines enclose the different compartments of the model: Proximal tear (PT), false lumen (FL), true lumen (TL), distal tear (DT) and peripheral (PH) bed.

Aortic dissection is a cardiovascular disease caused by the formation of intimal tears in the aortic wall. The constant action of pulsatile pressure may separate the wall layers within the media as a consequence. Subsequently, the lumen is divided into two lumina separated by the intimal flap: the true (TL) and false lumen (FL), which communicate through tears. The TL is the aortic primitive lumen while the FL is the passage enclosed by the dissected layers (Fig 1a).

Despite success of acute treatment of descending aortic dissections (DADs) and advances in this field, patient follow-up continues showing a high number of late complications and mortality after surgery or medical treatment [1, 2], including FL aneurysmal dilatation, eventually leading to rupture [3].

Aortic wall elasticity is, besides haemodynamics, a variable often altered in DAD and potentially involved in long-term outcome [4, 5]. Nevertheless, it is still mostly not considered, since it is seldom assessed in clinical practice/in-vitro studies. Moreover, as far as we are concerned, current in-silico studies are only based on rigid-wall simulations [6, 7, 8], originating from studies in mono-luminal aortas and under the assumption that elasticity may have minor effect on the haemodynamic parameters analysed [9]. This is done in order to simplify the computational and modelling approach, since a fluid structure interaction (FSI) simulation in aortic dissections is far more difficult to implement than in the single luminal case and accurate local material properties of the wall are often not available and difficult to obtain. However, comparing our previous findings on rigid and compliant models [10, 11], flow direction across tears and along the cardiac cycle, as well as intraluminal pressures, do seem to be significantly influenced by wall elasticity. Furthermore, in our clinical observations, flow at both the proximal and distal tear is directed towards the FL in systole (even in the absence of significant side branches), which is impossible with a fully rigid wall. Therefore, the rigid-wall assumptions made when simulating mono-luminal aortas and aortic aneurysms are not valid anymore when a second lumen is present due to a dissection, acting as a side chamber rather than a parallel tube.

The aim of this study is to contribute to the understanding of haemodynamic and biomechanical phenomena relevant for the long-term of DAD by means of a lumped-parameter model. Lumped-parameter models help to recreate and understand several flow aspects of a system (including the effects of wall elasticity), minimizing the need for complex in-silico, in-vivo or in-vitro experiments. Compared to these approaches, lumped-parameter models are able to quantitatively and qualitatively describe extensive pressure and flow waveforms without providing detailed solution on, mainly, local phenomena. They do provide a reasonable initial means to assess the overall system behaviour, and have a great potential to perform fast, easy and scalable studies on the influence of individual parameters [12].

In this study we have improved the first simplified version of our lumped-parameter model [10] with regards to its mathematical formulation, calibration and validation. The proposed model was calibrated and validated using experimental data. Next, it was used to study in more detail the effects of elasticity on intraluminal pressures and flow patterns across the tears. The results of this study highlight the fact that considering wall elasticity leads to clear differences in intraluminal heamodynamics in DAD.

## Materials and Methods

### Anatomic scenarios

A DAD was modelled as two parallel channels: TL (0.008m inner radius; 0.002m wall thickness; 0.16m length) and FL (0.01615m inner radius; 0.001m wall thickness; 0.16m length) communicated by holes to represent tears (Fig 1b). The FL radius was chosen so that the area of the circular FL in the numerical model corresponds to the experimental FL area where the FL is enclosing the TL (Fig 1c). We modelled 8 anatomic scenarios based on the possible permutations of varying tear size (4/10 mm diameter), number (1/2) and location (proximal/distal of the dissected region), which provides a good spectrum to validate our model. The notation *S*_*PROXIMAL SIZE*,*DISTAL SIZE*_ is used for designing each scenario where the subscript 0 denotes absence of a tear.

### In-vitro experimental model

Data from our previous in-vitro study [13] were used for building and validating the model.

Briefly, DAD was modelled as a physical phantom (Fig 1b and 1c) of compliant material where FL and TL were communicating via 4/10 mm diameter holes, mimicking clinically considered small and large tears, respectively, which was connected to a dynamic fluid circuit.

TL and FL pressures were measured at the proximal and distal sites of the phantom using retrograde catheterization with a pressure transducer (SPC-350 5F, Millar Instruments, TX, USA.) Velocities across tears were monitored using pulsed-wave Doppler echocardiography. Inlet flow waveforms were measured 0.15 m proximal to the dissected segment using a flow probe (Transonic Systems Inc, NY, USA). Pressure and flow waveforms were recorded using a PowerLab 16/30 together with LabChart Pro software (ADInstruments, Colorado Springs, CO, USA). The perfusion fluid was water at 25°C.

A detailed description of the phantom and the circuit can be found in Rudenick et al. [13].

### Mathematical formulation of the lumped-parameter model

A lumped-parameter model of a DAD was developed to recreate intraluminal haemodynamics (Fig 1d). Only the dissected region was represented where TL and FL were modelled as parallel compartments connected by resistances to mimic rigid tears.

The formulation of the model was mainly based on a lumped-parameter description of the blood flow in a compliant cylindrical vessel [12, 14]. The mathematical description is given by the simplification and averaging of the Navier-Stokes equations for an incompressible fluid and the introduction of the electrical-network analogy of these equations.

Following this analogy, each lumen of the dissected segment was modelled as an individual compartment using a L-type network where the components were the local resistance to flow (*R*_*LUMEN*_), compliance of the lumen (*C*_*LUMEN*_) and intraluminal inertial properties of flow (*L*_*LUMEN*_).

The peripheral connection was represented by a pure resistance (*R*_*PH*_) to describe the systemic vascular bed and was computed by dividing the experimental mean outlet pressure by the corresponding mean outflow. Since the mathematical model was calibrated to the experimental one (which ended with a resistive valve and a long, rather stiff, PVC tube), using a pure resistance at the periphery of the mathematical model did not have major influence on the final solution compared to a 3-element Windkessel model.

Proximal and distal tears were modelled as rigid entities by resistances *R*_*PT*_ and *R*_*DT*_, respectively.

The electrical components of each lumen were computed following Eqs 1–3, where *l* and *r* are lumen length and radius; *μ* and *ρ* represent the fluid dynamic viscosity (8.9E-4 Pa s) and density (997.0479 kg m^-3^); *E* the wall Young’s modulus; and *h* the wall thickness.

$$R_{lumen}=\frac{8\mu l}{\pi r^{4}}$$ (1)

$$L_{lumen}=\frac{\rho l}{\pi r^{2}}$$ (2)

$$C_{lumen}=\frac{3\pi r^{3}l}{2Eh}$$ (3)

In the TL, since the upstream flow (Q_TLi_) is known and assuming that the downstream pressure (P_PH_) is given, the upstream pressure is governed by:

$$\frac{dP_{TL}}{dt}=\frac{Q_{TLi}-Q_{TLo}}{C_{TL}}$$ (4)

and the downstream flow rate is:

$$\frac{dQ_{TLo}}{dt}=\frac{P_{TL}-P_{PH}-R_{TL}Q_{TLo}}{L_{TL}}$$ (5)

A similar reasoning is followed for modelling the FL (assuming known upstream flow (Q_PT_) and downstream pressure (P_DT_)) where Eqs 6 and 7 define the upstream pressure and the downstream flow, respectively:

$$\frac{dP_{FL}}{dt}=\frac{Q_{PT}-Q_{FL}}{C_{FL}}$$ (6)

$$\frac{dQ_{FL}}{dt}=\frac{P_{FL}-P_{DT}-R_{FL}Q_{FL}}{L_{FL}}$$ (7)

Since tears and peripheral connection are modelled as pure resistances, following Ohm’s law, the flow at the proximal tear is given by:

$$Q_{PT}=\frac{P_{TL}-P_{FL}}{R_{PT}}$$ (8)

upstream pressure at the distal tear is:

$$P_{DT}=R_{DT}Q_{FL}+P_{PH}$$ (9)

and upstream pressure at the peripheral connection is:

$$P_{PH}=R_{PH}Q_{PH}+P_{o}$$ (10)

Finally, based on Kirchhoff’s junction rule, flows at the TL inlet and at the end junction of both lumina are:

$$Q_{TLi}=Q_{i}-Q_{PT}$$ (11)

$$Q_{PH}=Q_{FL}-Q_{TLo}$$ (12)

The resultant system of differential algebraic equations was numerically solved with Matlab (MathWorks, Natick, MA) using the function ODE15s (time step: 0.01s). The solver was iterated until a steady state.

### Estimation of the model parameters for the experimental scenarios

The values of most of the components of the model were computed from geometric and haemodynamic data using Eqs 1–3. However, since the Young’s moduli of the phantom lumina were unknown and the velocity profiles at the tears were not parabolic, values of *C*_*TL*_, *C*_*FL*_, *R*_*PT*_ and *R*_*DT*_ were estimated via fitting the model to the experimental data using the Matlab implementation of the Nelder-Mead simplex direct search algorithm (convergence criteria of 1e-6) (Table 1). The fitting algorithm optimised the sum of the root mean square errors between the predicted and the experimental TL and FL pressures waveforms, at the distal and proximal tears. A preliminary parameter study was firstly conducted to determine the valid range of values for each parameter to estimate their initial values.

**Table 1:** Estimated parameters’ values of the lumped-parameter model.

Parameter	Value
**R**_**PT**_ **,R**_**DT**_ **(mmHg (ml s** ^**-1**^ **)** ^**-1**^ **)**	
Small tear	2.2200
Large tear	0.1434
**E**_**TL**_ **(MPa) / C**_**TL**_ **(ml mmHg** ^**-1**^ **)**	
1.07 / 0.0016	
**E**_**FL**_ **(MPa) / C**_**FL**_ **(ml mmHg** ^**-1**^ **)**	
S_4,0_	3.82 / 0.1110
S_10,0_	2.41 / 0.1760
S_0,4_	2.97 / 0.1427
S_0,10_	1.55 / 0.2735
S_4,4_	4.19 / 0.1011
S_4,10_	2.86 / 0.1480
S_10,4_	1.51 / 0.2813
S_10,10_	2.49 / 0.1700

*PT*: proximal tear; *DT*: distal tear; *TL*: true lumen; *FL*: false lumen; *PH*: peripheral

Some assumptions were made for the parameter estimation. Since a different phantom was used for each scenario and the FL latex piece was custom made, the elasticity of these pieces could differ from one model to another due to thickness variations resulting from their making process. Therefore, we estimated the Young’s modulus of the FL wall for each model in order to estimate its compliance. On the other hand, since the TL was made out of a standard silicone tube, the Young’s modulus of the TL wall was estimated and fixed for all cases. A similar approach was used for the resistance value of a small and a large tear. Under the previous assumptions, at first, a simultaneous fitting was performed for cases *S*_*0*,*4*_ and *S*_*0*,*10*_ with the same model variables, except for the resistances at the tears and FL wall elasticity. This first step provided a common value for TL compliance, the reference resistance values for a small and a large tear, and the FL compliance for each model. Afterwards, only the FL wall elasticity was fitted for the rest of the cases while fixing TL wall elasticity and tear resistances with the previously predicted values. Thus, we got a common dataset of parameters for all experimental scenarios, except for FL wall elasticity that was assumed to differ from one scenario to another.

### Model validation

Firstly, the mathematical model was used to simulate 8 different experimental scenarios where numerical predictions could be compared against experimental results. The corresponding experimental inflow waveform was imposed at the inlet and a venous zero-pressure was imposed at the outlet in all cases.

We quantified the goodness of fit by computing the relative root mean square error (rRMSE) between predicted and experimental pressure waveforms close to the tears. A qualitative comparison was performed between predicted velocities profiles across the tears and the counterpart pulsed-wave Doppler measurements. For each scenario, we also compared predicted and experimental input impedances (Zins). Zin was computed as the complex ratio of corresponding pressure and flow harmonics. Magnitude and phase angle were computed for the first 10 harmonics.

### Simulation of elasticity variations

Finally, the model was used to assess the effects of changes in wall elasticity on pressures and flow patterns through the analysis of several haemodynamic variables: a) proximal and distal TL (SP_TL_) and FL (SP_FL_) systolic pressure; b) proximal and distal TL (DP_TL_) and FL (DP_FL_) diastolic pressure; c) proximal and distal TL (PP_TL_) and FL (PP_FL_) pulse pressure (PP_LUMEN_ = SP_LUMEN_—DP_LUMEN_; LUMEN = TL/FL); d) pressure gradient across tears assessed through the computation of the false lumen systolic/diastolic pressure index (FPI_systolic/diastolic_%) as a percentage of TL systolic/diastolic pressure [13]; e) time shifting of proximal and distal FL pressure waveform with respect to the corresponding TL pressure waveform (TSF = time of SP_FL_—time of SP_TL_); f) quantification of change in direction between flows at the proximal and distal tears through the index of direction (*ID* = |*Q*_*PT*_+*Q*_*DT*_|/(|*Q*_*PT*_|+|*Q*_*DT*_|); PT = proximal tear; DT = distal tear). Values range between 0 and 1, where a value 1 corresponds to proximal and distal flows moving in the same direction along the lumina.

The analysis was conducted on scenarios *S*_*4*,*4*_ and *S*_*10*,*10*_, which were taken as reference cases, most often present in clinical practice [15]. For both scenarios, the Young’s moduli of the lumina’s walls (E^ref^) resulting from the calibration to the experimental models, were simultaneously changed by a factor of 0.35 to 1e7, so that wall Young’s modulus ranged from the one corresponding to a 20/30-year-old healthy individual (approx. 0.4 MPa) [16, 17] to a rigid wall’s (approx. 1e7 MPa).

## Results

### Mathematical versus experimental model

**Fig 2:**
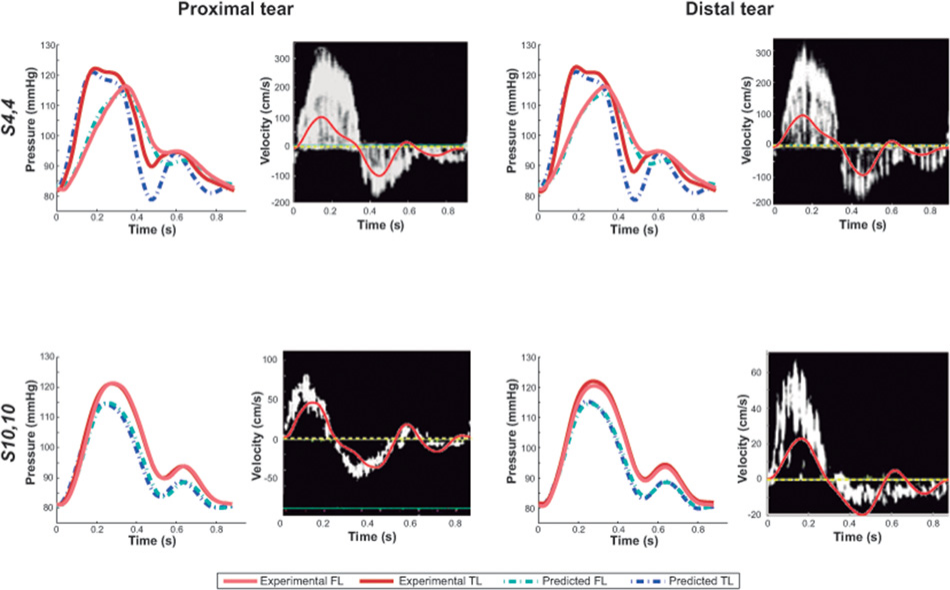
Experimental versus predicted intraluminal pressures and velocities across the tears. Comparison at the proximal and distal sites of the model, for scenarios *S_4,4_* and *S_10,10_*. Doppler positive velocities are directed from the TL to the FL and negative velocities the other way around.

We found an overall good agreement in both profile and values, between predicted and experimental TL/FL pressures, for all experimental scenarios at both proximal and distal tears. As shown in Fig 2, S1 Fig and Table 2, the overall predicted waveforms were close to the measured ones and rRMSEs for pressure were below 10%. In S2 Fig, TL and FL flow waveforms are additionally shown.

**Table 2:** Relative root square mean error (rRMSE) between predicted and measured pressures at the proximal and distal tears, for each scenario.

rRMSE(%)	Scenario							
	*S*_*4*,*0*_	*S*_*10*,*0*_	*S*_*0*,*4*_	*S*_*0*,*10*_	*S*_*4*,*4*_	*S*_*4*,*10*_	*S*_*10*,*4*_	*S*_*10*,*10*_
**Proximal tear**								
TL pressure	3.70	7.0			5.46	6.27	3.17	6.28
FL pressure	9.09	5.75			2.02	5.74	2.27	5.52
**Distal tear**								
TL pressure			2.65	1.29	4.95	8.09	2.19	6.27
FL pressure			5.02	1.83	1.73	6.61	1.52	5.02

*TL*, True lumen; *FL*, False lumen

When qualitatively comparing predicted (= mean flow in the tear) with Doppler flow velocities (= spectrum of all velocities present in the tears) across the tears (Fig 2, S1 Fig), there was an overall satisfactory agreement with the largest discrepancies observed at the small tears. The mathematical predictions reproduced the overall behaviour of experimental waveforms and generally there was a good quantitative agreement.

The pattern of Zin was similar between the numerical simulations and the experimental cases (S3 Fig), where the model Zin gives a reasonable overall estimate of the experimentally measured Zin, for both moduli and phase angles. While there was overall good agreement, the model does not fully represent the oscillations seen on the experimental impedance modulus and phase, because the numerical model does not exactly describe high frequency details such as inflection point and elevation in pressures [18]. However, the inlet pressure corresponding to each scenario has a power spectrum concentrated at the low frequencies (S3 Fig) where most of the signal information is found.

From this, we can conclude that the predictive capability of our model is satisfactory.

### Changes in wall elasticity

#### Pressures

**Fig 3:**
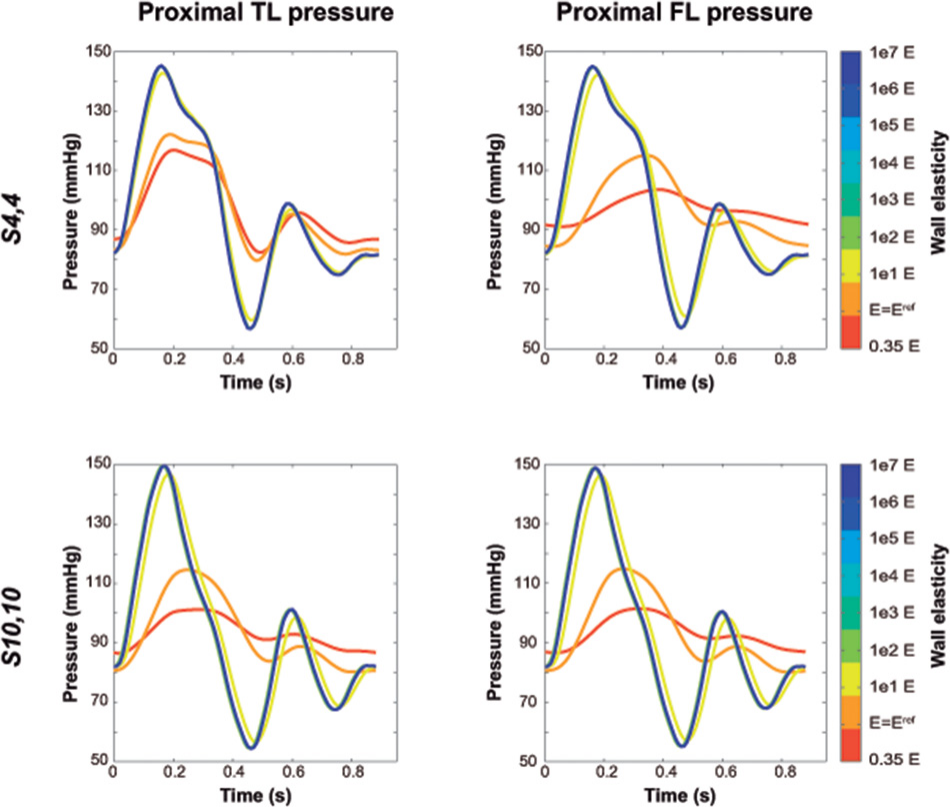
Changes in intraluminal pressures with changes in Young’s modulus. Variations in predicted intraluminal true (TL) and false lumen (FL) pressures, close to the proximal tear, with changes in Young’s modulus, for scenarios *S_4,4_* and *S_10,10_*. The value of *E = E^ref^* corresponds to the reference Young’s modulus of the lumen wall, resulting from the calibration of the computational model to the experimental one. Intraluminal pressures did not show substantial differences when the Young’s modulus was increased more than 1e2 *E*.

**Fig 4:**
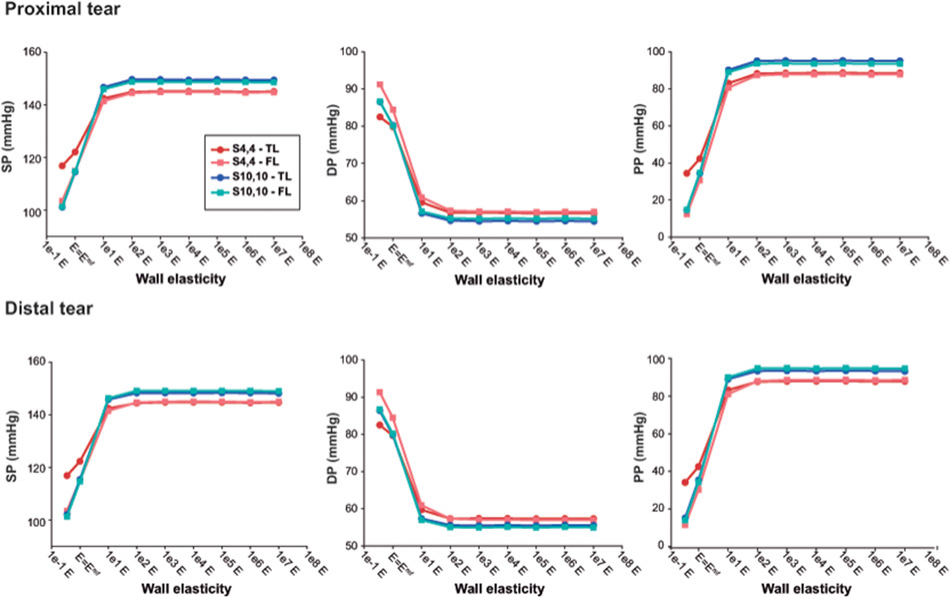
Intraluminal pressure indexes with changes in Young’s modulus. Values of predicted true (TL) and false lumen (FL) systolic pressure (SP), diastolic pressure (DP) and pulse pressure (PP), computed for different values of Young’s modulus for scenarios *S_4,4_* and *S_10,10_*. The value of *E = E^ref^* corresponds to the reference Young’s modulus of the lumen wall, resulting from the calibration of the computational model to the experimental one.

**Fig 5:**
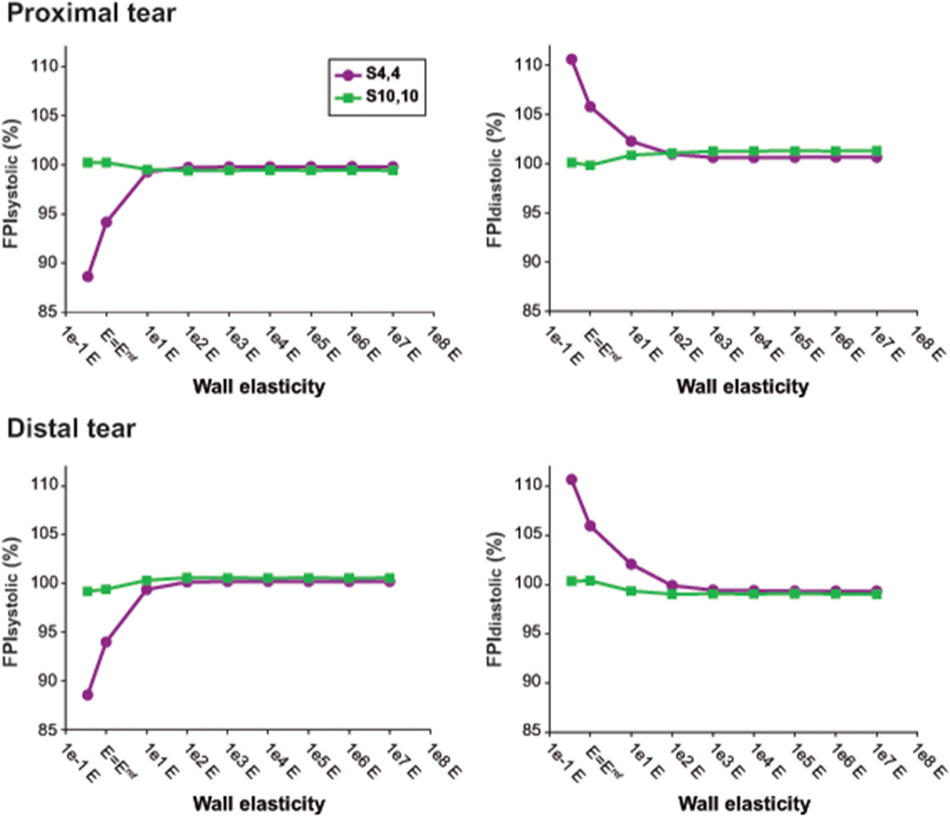
Pressure gradients across the tears with changes in Young’s modulus. Variations in predicted false lumen systolic (FPI_systolic_%) and diastolic pressure (FPI_diastolic_%) indexes with changes in Young’s modulus, at the proximal and distal tears for scenarios *S_4,4_* and *S_10,10_*. The value of *E = E^ref^* corresponds to the reference Young’s modulus of the lumen wall, resulting from the calibration of the computational model to the experimental one.

**Fig 6:**
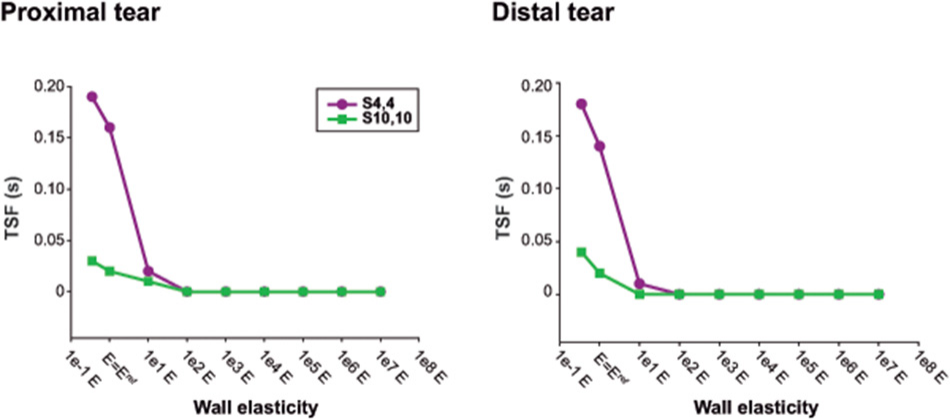
Time shifting variations of false lumen waveforms with changes in Young’s modulus. Time shifting experienced by the predicted false lumen pressure waveform in comparison with the true lumen pressure waveform (TSF) for the different values of Young’s modulus, at the distal and proximal sites of the dissected model. The value of *E = E^ref^* corresponds to the reference Young’s modulus of the lumen wall, resulting from the calibration of the computational model to the experimental one.

Independent from location (distal/proximal), a lower stiffness was associated with more damped TL and FL pressure curves (Fig 3), with lower SPs, higher DPs, and thus lower PPs (Fig 4). As the wall became stiffer, TL/FL pressure gradients across the tears decreased, so that FL SPs increased and FL DPs decreased approaching corresponding TL pressures, with resultant values of FPI_systolic_% and FPI_diastolic_% close to 100% (Fig 5). This effect was more pronounced for scenario *S*_*4*,*4*_ where TL/FL pressure gradients at the reference configuration were larger than in scenario *S*_*10*,*10*_ (Proximal FPI_systolic_%: 94.2% vs 100.2%; Proximal FPI_diastolic_%:105.8% vs 99.8%). In the presence of a low stiffness, FL pressure waveforms arrived later at both proximal and distal locations compared to TL pressure curves (Fig 6) while when stiffness was increased, time delay of FL pressure waveforms decreased until zero for the most rigid scenarios, where TL and FL curves overlapped.

#### Flows

**Fig 7:**
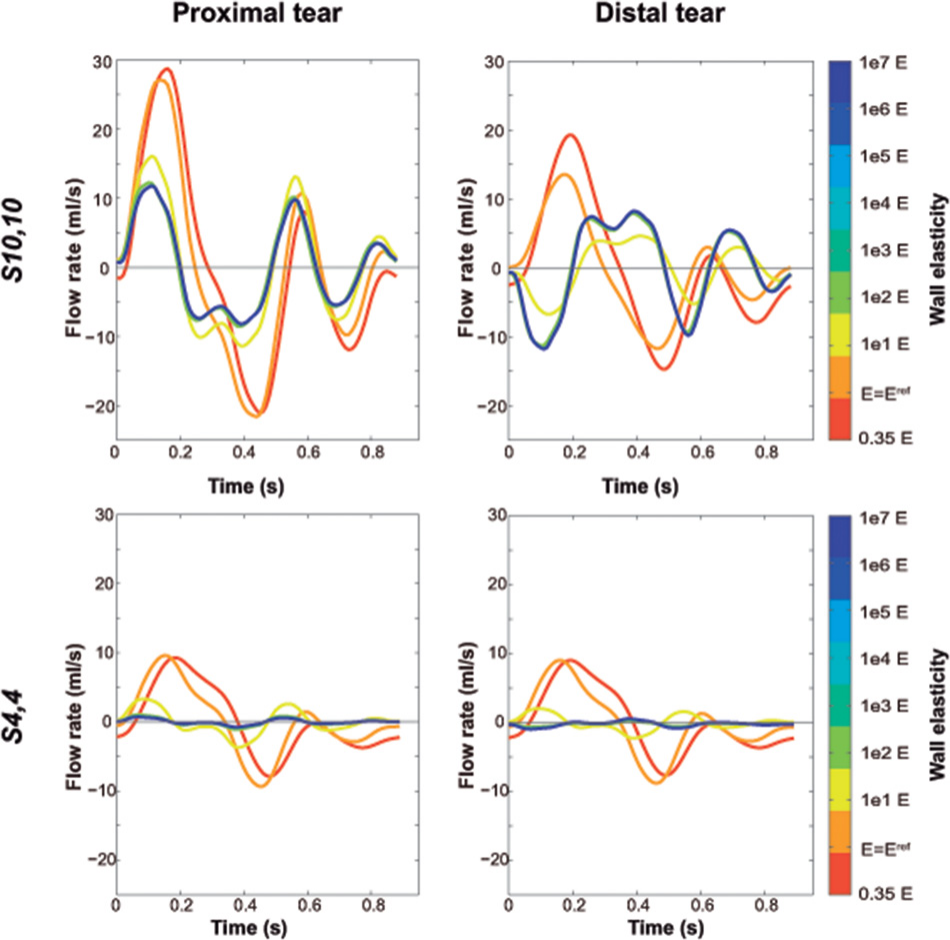
Changes in flow waveforms across the tears with changes in Young’s modulus. Variations in predicted flow waveforms across the proximal and distal tears with changes in Young’s modulus, for scenarios *S_4,4_* and *S_10,10_*. Positive flow rate corresponds to flow from the true lumen towards the false lumen. The value of *E = E^ref^* corresponds to the reference Young’s modulus of the lumen wall, resulting from the calibration of the computational model to the experimental one. Flow waveforms did not show substantial differences when the Young’s modulus was increased more than 1e2 *E*.

**Fig 8:**
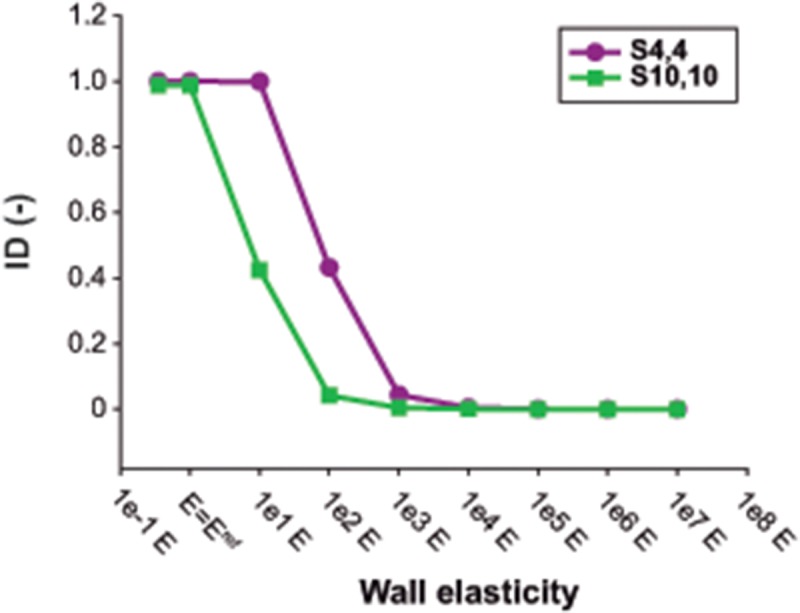
Changes in flow direction across the tears with changes in Young’s modulus. Index of direction (ID) computed for different values of Young’s modulus for scenarios *S_4,4_* and *S_10,10_*. The ID quantifies the change of direction between the flows across the proximal and distal tears, so that high ID values mean proximal and distal flows simultaneously moving from the true lumen to the false lumen or vice versa. The value of *E = E^ref^* corresponds to the reference Young’s modulus of the lumen wall, resulting from the calibration of the computational model to the experimental one.

Fig 7 displays the effect of Young’s modulus on flow waveforms across the tears. In the presence of low wall stiffness, the FL behaved as a side chamber of the TL, so that during the cardiac cycle flow went into or out of the FL simultaneously at proximal and distal tears. On the other hand, as wall stiffness increased, TL and FL acted as parallel compartments, so that flow entering the FL at the proximal tear at the same time went out the FL from the distal tear and vice versa. This phenomenon can be better appreciated through the assessment of the ID (Fig 8), which decreased with increasing stiffness until becoming zero. The effect was also more evident for case *S*_*10*,*10*_, where intraluminal communications are larger than in case *S*_*4*,*4*_ and thus, more flow is passing through the tears.

Additionally, decreased wall stiffness was associated with flow waveforms across the tears with higher amplitude, time-delayed peak flow and increase inflow reversal at both lumina (Fig 7).

## Discussion

A detailed knowledge of the flow phenomena in DADs is of importance in diagnosis and better understanding of their chronic development and clinical outcome. The main scope of this study was the development/validation of a lumped-parameter model of a DAD as a first simple approach for the characterization of intraluminal pressures and flows and study the influence of e.g. wall elasticity on flow directions and pressure elevations without a need for capturing detailed local flow phenomena. This has the advantage that it allows assessment of individual factors affecting global pressures and flows in a more feasible and scalable way than could be performed by complementary complex in-vivo, in-vitro and in-silico approaches. The model was validated with previous experimental in-vitro scenarios and was in turn used to assess the effects of wall elasticity variations on intraluminal pressures and flows.

Overall, a good agreement was found between the model-based predictions and experimental measurements. The proposed model recreated experimental pressure and velocity measurements for the different scenarios. Instantaneous values and profiles of predicted intraluminal pressures were consistent with the in-vitro approach, showing an rRMSE less than 10% for all cases.

Overall, qualitative features of velocity waveforms through tears were also in good agreement, keeping in mind that spectral Doppler measures the whole range of velocities within the sample volume at each instant of time (with the envelope corresponding to the maximal velocity in the centre of the flow profile) whereas only the instantaneous mean velocity is provided by the simulations. Large tears have a flat profile (Womersley’s parameter approx.: 12.5–13) and so a spectral Doppler with a narrow range of velocities while small tears develop a more parabolic velocity profile (Womersley’s parameter approx.: 5) and thus a much broader Doppler range of velocities. Taking into account these considerations, the predicted velocity profiles across tears were comparable with pulsed-wave Doppler measurements at all tears.

The similarities between the experimental and predicted Zin gave also strong evidence of the robustness of the model to recreate experimental results and its validity to be used as a complementary approach.

The model allowed studying the effects of properties that have not been studied before in DAD. Arterial elasticity is a biomechanical property with an important influence on arterial haemodynamics and thus clinical evolution, since it has clear effects on pressures and WSS [19, 20].

Our model shows that wall elasticity had major effects on flow patterns through tears. When wall elasticity was low enough, TL and FL behaved as parallel chambers, so that flow was one-way, simultaneously displacing fluid in both lumina from the proximal to the distal site and vice versa during the cardiac cycle. However, when wall elasticity was increased, tear flow dynamics completely changed and both proximal and distal tears simultaneously behaved as entry and exit sites. This additionally introduced significant flow reversal in the different compartments of the dissections, a phenomenon often seen in clinical practice [21]. The scenario where both tears act as entry and exit sites simultaneously during a cardiac cycle could be a potential cause of simultaneous jets getting into the FL from several locations and the consequent presence of disturbed flows and WSS variability. This flow behaviour was previously observed when comparing our computational rigid-wall simulations [22] and in-vitro experiments [13] and was one of the stimuli for the present study. The results are also in agreement with Tan et al. [23], where turbulence intensity was significantly higher in a compliant model in comparison with a rigid model of a thoracic aortic aneurysm.

Wall elasticity also had clear effects on intraluminal pressures. Diminished elasticity resulted in FL pressure waves of higher amplitude with higher SP, lower DP and resultant higher PP, so that FL pressure profiles approached TL’s, affecting TL/FL gradients. In the context of DADs, this might be associated with FL expansion and TL narrowing [24, 25], both potential complications during the long-term follow-up [26].

The majority of 3D in-silico flow studies in the field of aortic diseases are based on rigid-wall assumption, under the assumption that the effect of wall elasticity on the quantitative results is rather limited for the haemodynamic parameters studied [1]. However, our findings showed that elasticity appears to be extremely relevant in the pressure and flow prediction of DAD, where 2 parallel lumina are present, which is in line with the study performed on the aorta by Reymond et al. [27]. Wall elasticity seemed to affect pressures or flows depending on the size of communications between the lumina. When communications were large enough, wall elasticity seemed to be important in flow pattern determination while when communications were small enough, wall elasticity played an important role in pressure prediction, as it is also shown in Soudah et al. [11].

Therefore, the inclusion of wall elasticity is clearly altering intraluminal haemodynamics compared to a rigid-wall simulation and should be taken into account when assessing and studying aortic dissections’ using computational modelling.

These initial results also improve our understanding of haemodynamics in aortic dissections and can be further extended with the implementation of FSI simulations, in order to assess the spatial distribution of flow patterns, pressures and derived clinical parameters of relevance, such as wall shear stresses, and study the effects of changes in morphologic configurations on lumen haemodynamics. Additionally, it suggests that flow direction and its changes during the cardiac cycle might be clinically relevant parameters to study in more detail in these patients and that the (direct or indirect) measurement of wall elasticity can provide further insight in an individual patient. A better understanding of these dynamics might be useful to identify the possible factors involved in FL aneurysmal growth and rupture. Additionally, this knowledge can suggest more aggressive blood pressure lowering therapy in certain patients as well as the assessment of long-term risks of therapeutic options, such as fenestration or endovascular stent grafting treatments.

In conclusion, the proposed model seems to be a good first approximation to assess flow and pressure waveforms in DAD. The model in turn was useful to support the hypothesis that elasticity is a key biomechanical property to be considered in the haemodynamic assessment of aortic dissections.

### Limitations

The model used is a lumped-parameter representation, considering the TL and FL as two interacting compartments. Although it provides pressure and flows at the inlets and outlets of these chambers incorporating time-shifts and waveform changes due to inertial and elasticity effects, it does not explicitly account for wave travel and reflections. By omitting wave phenomena, which plays a significant role in shaping waveforms, the model is unable to describe detailed wave features and so capture absolute values of pressures or wall shear stresses, both important when assessing local dilatation in DAD. However, it is efficient in predicting global pressure and flow variations in presence or absence of strong reflections, so the neglecting of wave phenomena does not change the conclusions of the study. The model is not also able to capture local flow phenomena induced by jets and turbulence which might determine the local or tortuous dilatation observed in patients. But again, the simplification still allows for capturing overall pressure changes, tear velocities and flow directions. We used a Poiseuille resistor to model each lumen which is a justified simplification for pulsatile flows in large arteries when studying global flow phenomena [28, 29].

Since it is a study of chronic DAD, approximations such as reduced flap motion, circular tears, and very enlarged FL are reasonable [30–33]. Moreover, the model corresponds to an idealised linear dissection with circular lumina, while in reality the scenario could be more complex, with the presence of tortuous lumina and helicoidal flaps. In addition, the model lacks abdominal side branches and does not account for the presence of any thrombus in the FL. However, while these simplifications might affect the resulting local flow complexity (and so resulting shear stress distributions and intraluminal pressures) it will not influence the global interluminal haemodynamics, which was the focus of this study. Moreover, the distal vascular bed has been modelled as a pure resistance in order to match the experimental results, when in reality the distal vascular bed is also compliant. However, if the distal vascular bed is described by a three element Windkessel model and the distal compliance is tuned to get pressures within the range of physiological values, using a compliant distal bed does not change the conclusions of the study. The generic model also seems to be ideal for performing an extense parametric study and to give a first insight into the role of wall elasticity in the determination of interluminal haemodynamics.

Predicted velocities across the tears were computed under the assumption that tear areas were reduced a 25% by catheter obstruction when performing retrograde catheterization in the in-vitro experiments. Pressures in the in-vitro model were measured at the level of the tears, close to the place where a high speed jet was registered. However, the transducer tip was carefully placed far enough from the jet to avoid as much as possible the depression of the registered static FL pressures. Moreover, the model was calibrated to fit experimental TL and FL pressures at the same time, which was not 100% realistic, since during the experiments tears were in turn obstructed by the catheter (a unique catheter was used) when measuring FL pressures by retrograde catheterization and velocities across tears were measured before performing catheterization. FL diameter and lumina’s thickness were also an approximation, since the physical experimental model does not have a perfect circumferential FL cross-section and uniform lumina’s thickness (Fig 1c). Therefore, these assumptions might be inducing some error in the predicted pressures and velocities.

## Supporting Information

S1 Fig Experimental versus predicted intraluminal pressures and velocities across the tears for all the scenarios. Comparison between experimental and predicted intraluminal pressures and velocities across the tears, at the proximal and distal sites of the model, for the eight experimental scenarios assessed. Doppler positive velocities are directed from the true lumen (TL) to the false lumen (FL) and negative velocities the other way around.
(EPS)

S2 Fig Predicted intraluminal flow profiles for all the scenarios. Predicted flow rates at the proximal and distal sites of the true lumen (TL) and false lumen (FL), for the eight experimental scenarios studied. Positive flows are antegrade and negative flows are retrograde.
(EPS)

S3 Fig Comparison between experimental and predicted input impedance for all the scenarios. Experimental and predicted input impedance (Zin) modulus (left) and phase (center), and power spectrum of the inlet pressure (right) computed for the eight anatomic scenarios studied.
(EPS)
